# Phenotypic and genomic analyses of the probiotic *Enterococcus casseliflavus* SHAMU-QH-02

**DOI:** 10.3389/fmicb.2025.1535506

**Published:** 2025-08-13

**Authors:** Xin-Yu Li, Ying Liu, Sheng-Nan Weng, Wei Chen, Gao Ju, Chuan-Yue Peng, Qiang Zhou, Jie Yao, Wei Tang

**Affiliations:** ^1^Department of Clinical Laboratory, The Second Affiliated Hospital of Anhui Medical University, Hefei, China; ^2^Key Laboratory of High Magnetic Field and Ion Beam Physical Biology, Hefei Institutes of Physical Science, Chinese Academy of Sciences, Hefei, China

**Keywords:** *Enterococcus casseliflavus*, probiotic, bacteriocin, antimicrobial, antioxidant, anti-inflammatory

## Abstract

The past few years have witnessed burgeoning interest in the potential beneficial role of probiotics in multiple fields. This study aimed to explore the probiotic properties and analyze the genomic information of the *Enterococcus casseliflavus* SHAMU-QH-02 strain, isolated from the human biliary tract. The SHAMU-QH-02 strain was identified using 16S rRNA gene and whole-genome sequencing. The strain exhibited antagonistic activity against most gram-positive cocci, gram-positive bacilli and gram-negative bacilli, especially foodborne pathogens, such as *Bacillus cereus*, *Listeria monocytogenes*, *Salmonella enteritidis*, *Vibrio fluvialis*, *Escherichia coli*, *Pseudomonas aeruginosa*, and *Staphylococcus aureus*. Antioxidant activity was assessed by ABTS^+^ scavenging assay and determination of ROS levels. Both the cell-free extract (CFE) and cell-free supernatant (CFS) of the SHAMU-QH-02 strain exhibited strong ABTS^+^ scavenging ability. Moreover, the CFS demonstrated a higher scavenging ability of ROS. Besides, the SHAMU-QH-02 strain could markedly decrease the production of interleukin-1β (IL-1β) in lipopolysaccharide (LPS)-stimulated RAW264.7 cells, indicating anti-inflammatory activity. Safety assessments indicated no cytotoxicity and susceptibility to 12 common antibiotics. Gastrointestinal stability assessment revealed high tolerance to intestinal pH and bile, yet limited ability to adhere to intestinal epithelial cells. Genomic analysis revealed the presence of hypothetical bacteriocin production genes (*n* = 2), virulence factor genes (*n* = 4), and antibiotic resistance genes (*n* = 23); however, none were located within the eight phage sequences. Importantly, the crude extract obtained using XAD-16HP resin could tolerate extreme pH values, 121°C, and multiple proteases. Taken together, the SHAMU-QH-02 strain exhibits probiotic attributes and presents as a notably promising probiotic candidate, potentially contributing to the food industry, health promotion and disease prevention.

## 1 Introduction

Probiotics play a key role in food science and clinical medicine, including the fermentation and preservation of foods, the development and utilization of functional foods, dietary supplements, and biopharmaceuticals (Sutton, 2008; Granato et al., 2010). Although probiotics have been extensively applied in various fields, there remains a need to develop novel, high-quality probiotics and related products (Cunningham et al., 2021).

Probiotics can inhibit or kill pathogens, regulate intestinal flora, and modulate the immune system, contributing to the treatment of cancer and metabolic disorders (Plaza-Diaz et al., 2019; Pereira and Kriegel, 2023). The probiotic metabolites, such as bacteriocins, organic acids, short-chain fatty acids, hydrogen peroxide, and exopolysaccharides, enhance the functional properties of probiotics. Among them, bacteriocin production is the most critical desirable trait in the selection of a probiotic strain (Dobson et al., 2012). The vast majority of bacteriocins are peptides or proteins synthesized by bacterial ribosomes, exhibiting bacteriostatic or bactericidal effects on other strains of the same or related species (Huang et al., 2021). Bacteriocins possess numerous desirable properties, including low toxicity, biodegradability, biocompatibility, and stability, as well as unique bactericidal mechanisms distinct from conventional antibiotics (Cotter et al., 2013; Walsh et al., 2021). Therefore, it is of great importance to screen and explore bacteriocin-producing probiotics.

*Lactobacillus* is a major producer of bacteriocins among probiotics, along with *Enterococcus*, *Lactococcus*, *Streptococcus*, *Pediococcus*, *Aerococcus*, *Alliococcus*, *Carnobacterium*, *Dolosigranulum*, *Oenococcus*, *Tetragenococcus*, *Vagococcus*, and *Weissella* (Bintsis, 2018). Among these, *Enterococcus* is increasingly investigated as a potential probiotic candidate, with *Enterococcus faecium* being commonly available as a probiotic on the market (Zommiti et al., 2018). Notably, *Enterococcus casseliflavus* strains have been previously reported to exhibit a narrow antimicrobial spectrum. Relevant studies have primarily focused on the isolation, purification, and antimicrobial mechanisms of the produced bacteriocins. There have been few reports on the probiotic functions of broad-spectrum bacteriocin-producing *E. casseliflavus*, especially antioxidant and anti-inflammatory properties.

In this study, we screened and patented the SHAMU-QH-02 strain, which was originally isolated from the human biliary tract. The probiotic properties and genomic information of SHAMU-QH-02 were explored.

## 2 Methods

### 2.1 Bacterial strains and culture conditions

The *E. casseliflavus* SHAMU-QH-02 strain used in this study was isolated from a human bile sample obtained from a patient undergoing treatment at the hepatobiliary pancreatic surgery department of the Second Affiliated Hospital of Anhui Medical University. The SHAMU-QH-02 strain has been deposited at the China Center for Type Culture Collection (CCTCC) under accession number CCTCC M 2023277. SHAMU-QH-02 was stored in 15% glycerol broth at −80°C and subcultured before being used for any experiment. SHAMU-QH-02 was grown in Luria-Bertani broth (LB; Sangon Biotech, Shanghai, China) under shaking at 220 rpm or on Columbia sheep blood agar plates (BAP; Tianda, Hefei, China).

All indicator strains were isolated from the respiratory tract, intestines, urethra, blood, skin, and infected tissues of clinical patients at the Second Affiliated Hospital of Anhui Medical University from 2021 to 2023.

### 2.2 Strain identification

SHAMU-QH-02 was identified by 16S rRNA gene sequencing and genome sequencing. The genomic DNA was extracted and subjected to PCR amplification using the bacterial universal primers 27F (5′-AGAGTTGATCCTGCTCAG-3′) and 1492R (5′-CTACGGCTACCTTGTTACGA-3′). The 16S rRNA gene sequencing and genome sequencing were completed by Shanghai Personalbio Corporation and Shanghai Sangon Biotech Corporation, respectively. A 16S rRNA gene-based phylogenetic tree and a whole-genome-based phylogenetic tree were constructed with NCBI BLAST software,^1^ MEGA 11.0 software,^2^ iTOL v7 software,^3^ and CVTree software (CVTree4; http://cvtree.online/v4/prok/).

### 2.3 Antimicrobial activity

Antimicrobial activity against various indicator strains was determined by the Oxford cup method, as previously described (Zhou et al., 2022). Briefly, each indicator strain (10^7^ CFU/mL) was spread on a LB agar plate using a sterile cotton swab. Once dry, 30 μL SHAMU-QH-02 suspension (10^9^ CFU/mL) was added into sterile Oxford cups (Φ = 6 mm). The plates were incubated at 30°C for 16 to 18 h, followed by the measurement of distinct inhibition zones around the cups to assess the antimicrobial activity.

### 2.4 Preparation of cell-free supernatant and cell-free extract

SHAMU-QH-02 was cultured in LB broth under shaking (200 rpm) at 35°C for 20 h. The broth culture was centrifuged at 7,547 g at 4°C for 5 min to separate the cell-free supernatant (CFS) and cell pellet. The CFS was harvested and sterilized by a 0.22 μm filter (Merck Millipore, Ireland). Subsequently, the aforementioned cell pellet was resuspended with phosphate buffer solution (PBS), followed by sonication for 10 min in an ice bath. The resulting supernatant was sterilized by a 0.22 μm filter and used as cell-free extract (CFE).

### 2.5 Antioxidant activity

#### 2.5.1 ABTS^+^ scavenging assay

The antioxidant activity of SHAMU-QH-02 was assessed by 2,2′-azino-bis (3-ethylbenzothiazoline-6-sulfonic acid) (ABTS), with slight modifications to the method described in the literature (Kruczek et al., 2023). Briefly, ABTS^+^ was generated by reacting ABTS stock solution (7 mM) and potassium persulfate (2.45 mM) after incubation at room temperature in the dark for 16 h. The ABTS^+^ solution was diluted to obtain an absorbance of 0.70 at 734 nm. Next, ABTS^+^ solution was added to different concentrations of the CFE and CFS of SHAMU-QH-02 (125 μg/mL or 500 μg/mL), mixed thoroughly and allowed to stand at room temperature for 10 min. Finally, the absorbance was measured at 734 nm using a microplate spectrophotometer (Spectra Max i3x, Molecular Devices, United States). The ABTS^+^ solution was used as the blank control. The scavenging ability was calculated by the following formula:

$$\begin{array}{l} {\text{ABTS}}^{+}\;\text{scavenging ratio}\;(\%)=\\ \frac{\text{Blank control}\;{\mathrm{OD}}_{734}-\text{Sample}\;{\mathrm{OD}}_{734}}{\text{Blank control}\;{\mathrm{OD}}_{734}}\times 100\% \end{array}$$

#### 2.5.2 Determination of ROS levels

Intracellular reactive oxygen species (ROS) levels were detected using the 2′,7′-dichlorodihydrofluorescein diacetate (DCFH-DA) assay, as described previously (Zhang et al., 2022). The mouse macrophage RAW264.7 cells (ATCC, Manassas, United States) density was adjusted to 10^5^ cells/mL, seeded into a 24-well Transwell plate (Costar, Corning, United States) at 1 mL per well. Subsequently, 25 μL, 50 μL, and 100 μL of SHAMU-QH-02 (OD_600_ = 0.6) and *E. coli* OP50 (OD_600_ = 0.6) suspension were seeded in the above 24-well Transwell plate at 37°C for 24 h. After centrifugation, the medium was discarded, and a 1:1,000 dilution of DCFH-DA probe (Beyotime, China) was added and incubated at 37°C for 30 min in the dark. Cells were washed three times with PBS to thoroughly remove extracellular DCFH-DA. Finally, the DCF fluorescence intensity was measured via a microplate spectrophotometer (Spectra Max i3x, Molecular Devices, United States) with excitation at 488 nm and emission at 525 nm. The medium with RAW264.7 cells served as the blank control.

### 2.6 Anti-inflammatory activity

The level of inflammatory cytokine IL-1β was determined by using ELISA kits (Cusabio, Wuhan, China) according to the manufacturer’s instructions. Briefly, the mouse macrophage RAW264.7 cells (10^5^ cells/mL) were seeded in a 24-well Transwell plate (1 mL/well), followed by incubation with LPS (100 ng/well). Next, 25 μL and 50 μL of SHAMU-QH-02 (OD_600_ = 0.6) and *E. coli* OP50 (OD_600_ = 0.6) suspension were seeded in the above 24-well Transwell plates at 37°C for 24 h. Successively, the plate was centrifuged, and the supernatant was collected. Finally, the absorbance was measured at 450 nm using a microplate spectrophotometer (Spectra Max i3x, Molecular Devices, United States). The medium with RAW264.7 cells served as the blank control.

### 2.7 Cytotoxicity assay

The cytotoxicity of SHAMU-QH-02 on mouse macrophage RAW264.7 cells was evaluated by the Cell Counting Kit-8 (CCK-8; Beyotime Biotech, Shanghai, China) assay according to the manufacturer’s instructions. Briefly, exponentially growing RAW264.7 cells (10^5^ cells/mL) were seeded in a 24-well Transwell plate (1 mL/well), followed by incubation with 25 μL, 50 μL, or 100 μL of SHAMU-QH-02 (OD_600_ = 0.6) suspension at 37°C for 24 h. Then, CCK-8 solution was added to the plate (10 μL/well) and incubated in the dark for 2 h. Finally, the absorbance was measured at 450 nm using a microplate spectrophotometer (Spectra Max i3x, Molecular Devices, United States). The cell culture medium without cells and bacteria served as a blank control.

$$\begin{array}{l} \text{The cell proliferation rate}\;(\%)=\\ \frac{\text{Bacteria}\;{\mathrm{OD}}_{450}-\text{Blank control}\;{\mathrm{OD}}_{450}}{\text{Cell}\;{\mathrm{OD}}_{450}-\text{Blank control}\;{\mathrm{OD}}_{450}}\times 100\% \end{array}$$

### 2.8 Antimicrobial susceptibility testing

Based on the broth microdilution method and the Clinical and Laboratory Standards Institute guidelines (CLSI M100-S29), antimicrobial susceptibility testing of SHAMU-QH-02 was performed using a VITEK 2 Compact System and a VITEK 2 AST-GP639 test kit (bioMerieux, France). Fourteen common antibiotics were tested, including vancomycin (VAN), tigecycline (TGC), teicoplanin (TEC), daptomycin (DAP), penicillin (PEN), ampicillin (AMP), ciprofloxacin (CIP), levofloxacin (LVX), tetracycline (TET), fosfomycin (FOS), erythromycin (ERY), linezolid (LNZ), nitrofurantoin (NF), and quinupristin/dalfopristin (QP/DP).

### 2.9 Gastrointestinal stability

#### 2.9.1 Acid–base and bile tolerance

Acid–base tolerance was conducted according to a previously described method (Cao et al., 2019; Chen et al., 2020). Briefly, the log-phase SHAMU-QH-02 cells were centrifuged and resuspended in sterile water to achieve a starting concentration of 10^8^ CFU/mL. Then, SHAMU-QH-02 suspension was diluted into aqueous solutions with different pH values (pH 2, 3, 4, 5, 6, 7, 8, and 9) or different porcine bile (0.6, 0.9, and 1.2%) to get a final concentration of 10^6^ CFU/mL. Following the incubation period, viable cell counts were performed on BAP agar plates at 0, 90, and 180 min. The experiments were performed in triplicate. The results were quantified as a percentage of viability relative to the control, calculated using the following equation.

$$\text{Tolerance}\;(\%)=\frac{\text{Number of viable cells}}{\text{Number of cells in control}}\times 100\%$$

#### 2.9.2 *In vitro* adhesion assay

The adhesion of SHAMU-QH-02 to intestinal epithelial cells was assessed using the Caco-2 epithelial cell line (ATCC, Manassas, United States). Briefly, Caco-2 cells were seeded at a density of 10^5^ cells per well in a 96-well flat-bottom microplate (Costar, Corning, ME, United States) without antibiotics and incubated at 37°C in a humidified, 5% CO_2_ incubator for 48 h. Two hundred microliters of SHAMU-QH-02 suspension (2 × 10^7^ CFU/mL) in Dulbecco’s modified Eagle’s medium (DMEM; Hyclone, UT, United States) was added to each well and allowed to incubate with Caco-2 cell monolayers at 37°C in a humidified, 5% CO_2_ incubator. After washing with PBS to get rid of non-adherent bacteria, Caco-2 cells were lysed with 0.1% (v/v) Triton X-100 and 0.1% (v/v) trypsin-EDTA (Sigma-Aldrich) for 15 min at 37°C. The serially diluted lysates were then plated onto BAP agar plates at 60 and 120 min to determine the number of adherent bacteria. The adhesion index was recorded as the percentage of adherent bacteria in bacteria added. The experiments were performed in triplicate.

### 2.10 Genome sequencing and sequence analysis

Genomic DNA was isolated from SHAMU-QH-02 using bead beating and phenol-chloroform extraction methods. The genome was sequenced using the ONT GridION platform (Oxford Nanopore Technologies Ltd., Oxford, United Kingdom) by Shanghai Sangon Biotech. The bacteriocin biosynthetic gene clusters were predicted by the Antibiotics and Secondary Metabolite Analysis SHell Database (antiSMASH; http://antismash.secondarymetabolites.org) and the Bayesian Analysis of Gene Essentiality (BAGEL; http://bagel.molgenrug.nl/). Virulence factors were recognized by querying the Virulence Factors of Pathogenic Bacteria Database (VFDB; http://www.mgc.ac.cn/cgi-bin/VFs/v5/main.cgi), and the antibiotic resistance genes were detected by the Comprehensive Antibiotic Resistance Database (CARD; https://card.mcmaster.ca/). The phage regions were identified by the Pathogen Host Interactions Database (PHI; http://www.phi-base.org/).

### 2.11 Stability of crude extract to pH, temperature, and proteases

The crude extract was obtained using a previously described method (Liu et al., 2021). Briefly, the CFS was loaded onto a column containing XAD-16HP resin (Source Leaf Biological Company, Shanghai, China) for 2 h. Subsequently, the resin was washed with 2 L of ddH_2_O, 1 L of 30, 40, 50, 60, 70, 80, and 90% (v/v) ethanol. Each gradient elute was collected and evaporated using a rotary evaporator, yielding a powder (crude extract). Afterward, each crude extract was dissolved in 5 mL of ddH_2_O, and its antimicrobial activity was tested by the Oxford cup method. The indicator strain used was *S. aureus* ATCC 25923.

The stability assay was performed using a previously described method with some modifications (Wang et al., 2021). Specifically, the crude extract solution was adjusted to pH levels of 3.0, 4.0, 5.0, 6.0, 7.0, 8.0, 9.0, 10.0, and 11.0 using 0.5 M NaOH and 0.5 M HCl solutions, followed by incubation at 35°C for 2 h. The residual antimicrobial activity was tested after neutralizing the sample to pH 7.0. Besides, the crude extract solution was exposed to 30°C, 40°C, 50°C, 60°C, 70°C, 80°C, 90°C, 100°C, and 121°C for 30 min, respectively. The residual antimicrobial activity was tested. Finally, the crude extract solution and proteases (20 mg/mL) were mixed in 1:1 (v/v) and incubated at 35°C for 2 h to achieve protease stability. The residual antimicrobial activity was determined. The proteases used included chymotrypsin, bromelain, trypsin, and proteinase K (Sigma Chemical Company, United States).

### 2.12 Statistical analysis

All statistical analyses were performed using IBM SPSS (v18.0; IBM Corp., Armonk, NY, United States). Statistical significance was defined as *p* < 0.05.

## 3 Results

### 3.1 Strain identification

**Figure 1:**
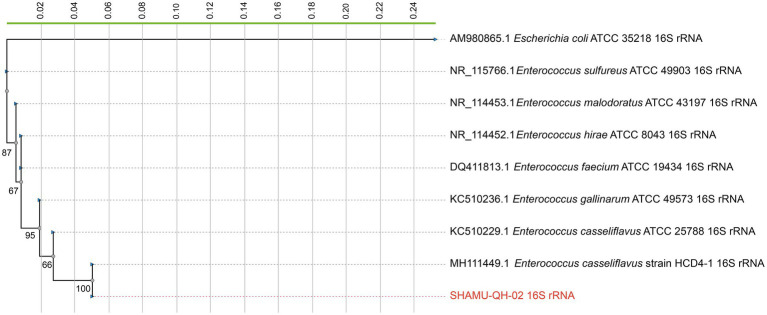
The phylogenetic tree constructed based on 16S rRNA gene sequences. SHAMU-QH-02 strain is shown in red. The scale bar indicates 0.02 substitutions per nucleotide position.

**Figure 2:**
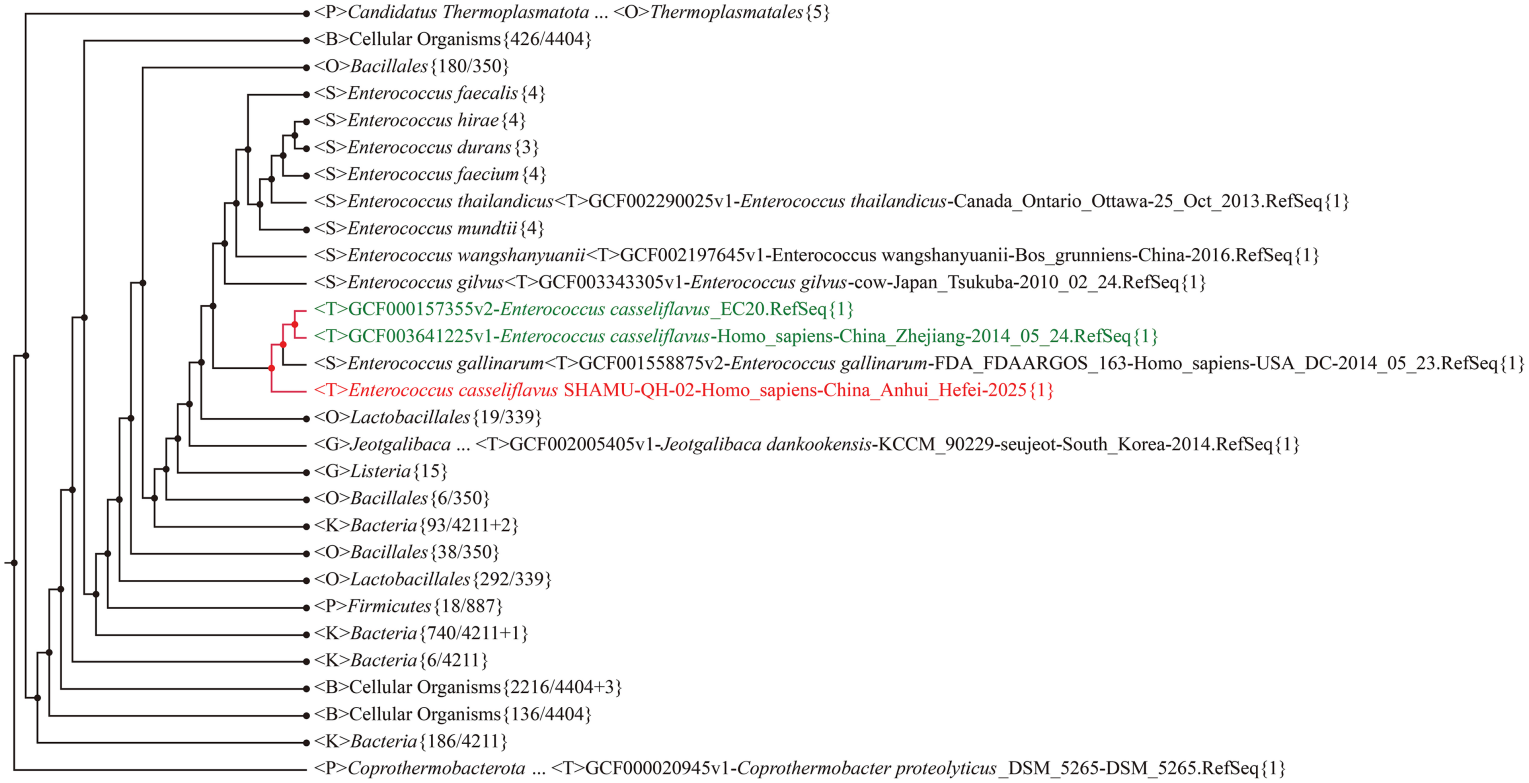
The phylogenetic tree based on whole-genome sequences. SHAMU-QH-02 strain is shown in red. Other *Enterococcus casseliflavus* strains are shown in green.

The 16S rRNA gene sequence of the SHAMU-QH-02 strain was deposited in GenBank (accession number: OQ918013). To further determine the strain, a 16S rRNA gene-based phylogenetic tree and a whole-genome-based phylogenetic tree (CVTree4) were constructed, respectively. Comprehensively, the SHAMU-QH-02 strain was identified as *Enterococcus casseliflavus*. Refer to Figures 1, 2 for more details.

### 3.2 Antimicrobial spectrum

**Figure 3:**
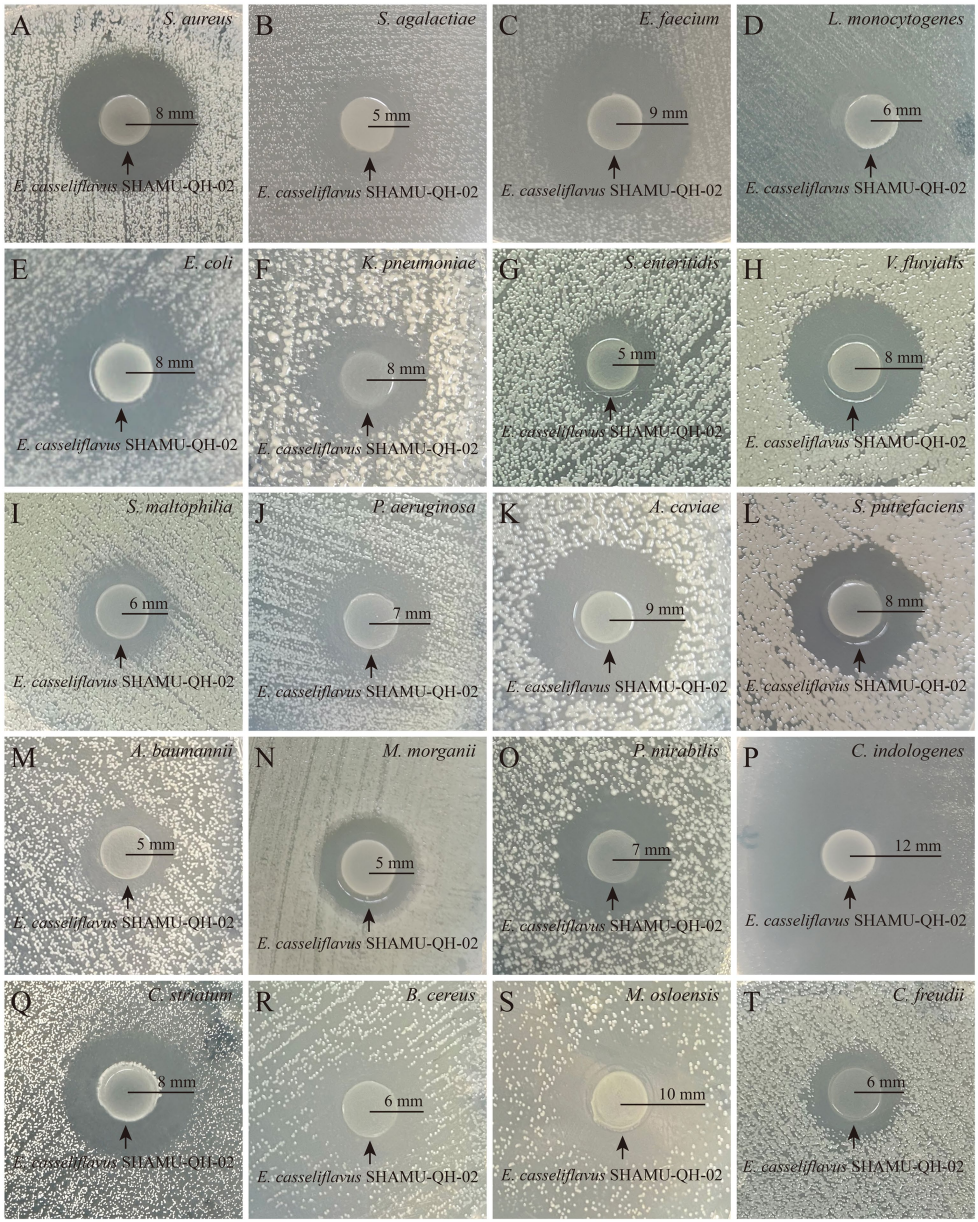
Antagonistic effect of SHAMU-QH-02 on the growth of various bacteria. **(A-T)** The inhibition zone diameters of SHAMU-QH-02 against *S. aureus, S. agalactiae, E. faecium, L. monocytogenes, E. coli, K. pneumoniae, S. enteritidis, V. fluvialis, S. maltophilia, P. aeruginosa, A. caviae, S. putrefaciens, A. baumannii, M. morganii, P. mirabilis, C. indologenes, C. striatum, B. cereus, M. osloensis,* and *C. freundii*.

SHAMU-QH-02 demonstrated antagonistic activity against the majority of gram-positive cocci, gram-positive bacilli, and gram-negative bacilli, including *Staphylococcus aureus* (Ф = 16 mm), *Streptococcus agalactiae* (Ф = 10 mm), *Enterococcus faecium* (Ф = 18 mm), *Listeria monocytogenes* (Ф = 12 mm), *Escherichia coli* (Ф = 16 mm), *Klebsiella pneumoniae* (Ф = 16 mm), *Salmonella enteritidis* (Ф = 10 mm), *Vibrio fluvialis* (Ф = 16 mm), *Stenotrophomonas maltophilia* (Ф = 12 mm), *Pseudomonas aeruginosa* (Ф = 14 mm), *Aeromonas caviae* (Ф = 18 mm), *Shewanella putrefaciens* (Ф = 16 mm), *Acinetobacter baumannii* (Ф = 10 mm), *Morganella morganii* (Ф = 10 mm), *Proteus mirabilis* (Ф = 14 mm), *Chryseobacterium indologenes* (Ф = 24 mm), *Corynebacterium striatum* (Ф = 16 mm), *Bacillus cereus* (Ф = 12 mm), *Moraxella osloensis* (Ф = 20 mm), *Citrobacter freundii* (Ф = 12 mm). Refer to Figure 3 for more details.

### 3.3 Antioxidative activity

#### 3.3.1 ABTS^+^ scavenging assay

**Figure 4:**
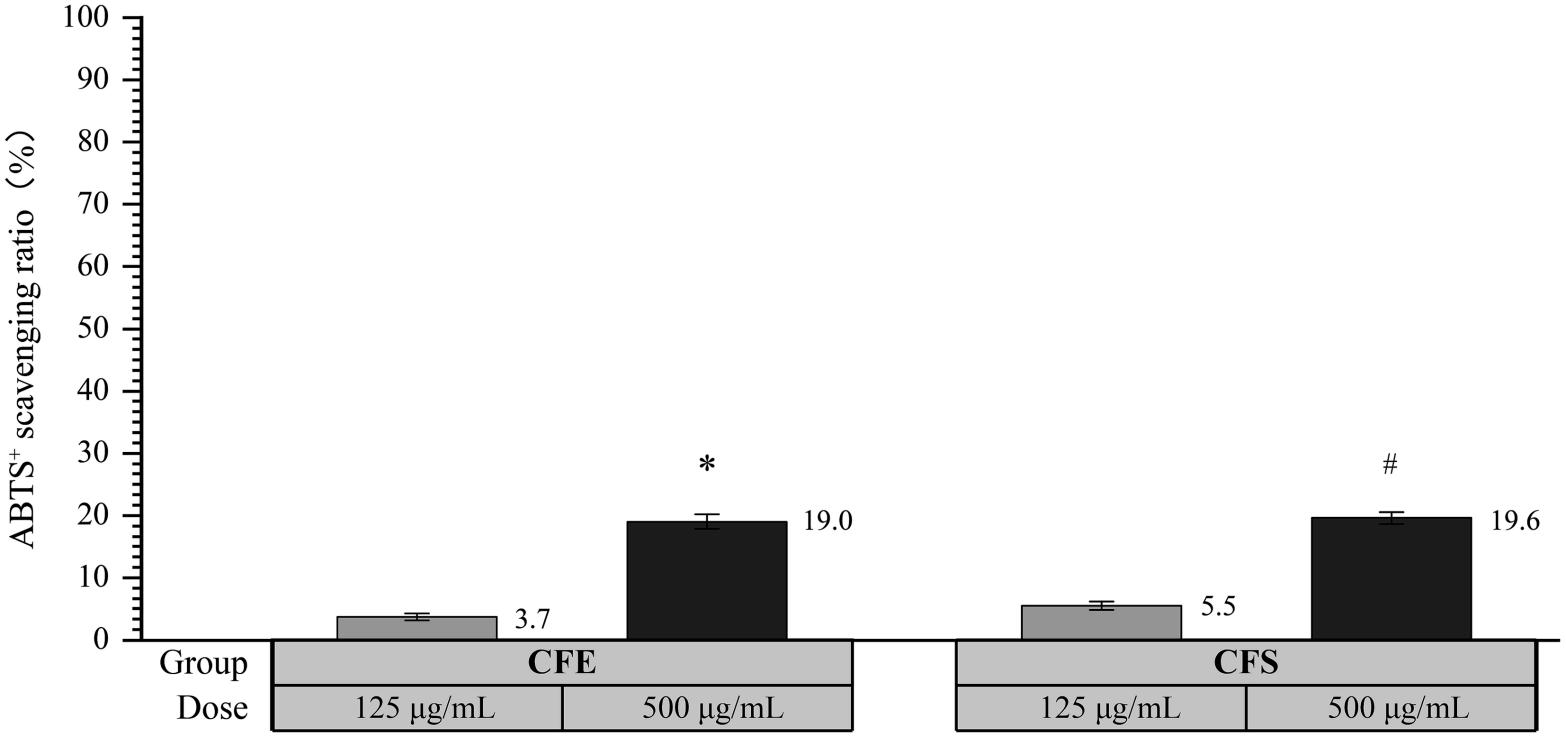
The antioxidant activity of SHAMU-QH-02 based on the ABTS^+^ assay. ^*^Statistically significant versus 125 μg/mL CFE. ^#^Statistically significant versus 125 μg/mL CFS.

As shown in Figure 4, the ABTS^+^ scavenging activity of CFE was 3.7 and 19.0% at concentrations of 125 μg/mL and 500 μg/mL CFE, respectively, demonstrating a statistically significant difference (*p* < 0.05). Moreover, the ABTS^+^ scavenging activity was 5.5 and 19.6% at concentrations of 125 μg/mL and 500 μg/mL CFS, respectively, exhibiting a statistically significant difference (*p* < 0.05). The above results suggest that both CFE and CFS derived from SHAMU-QH-02 exhibited strong ABTS^+^ scavenging ability in a dose-dependent manner.

#### 3.3.2 Determination of ROS

**Figure 5:**
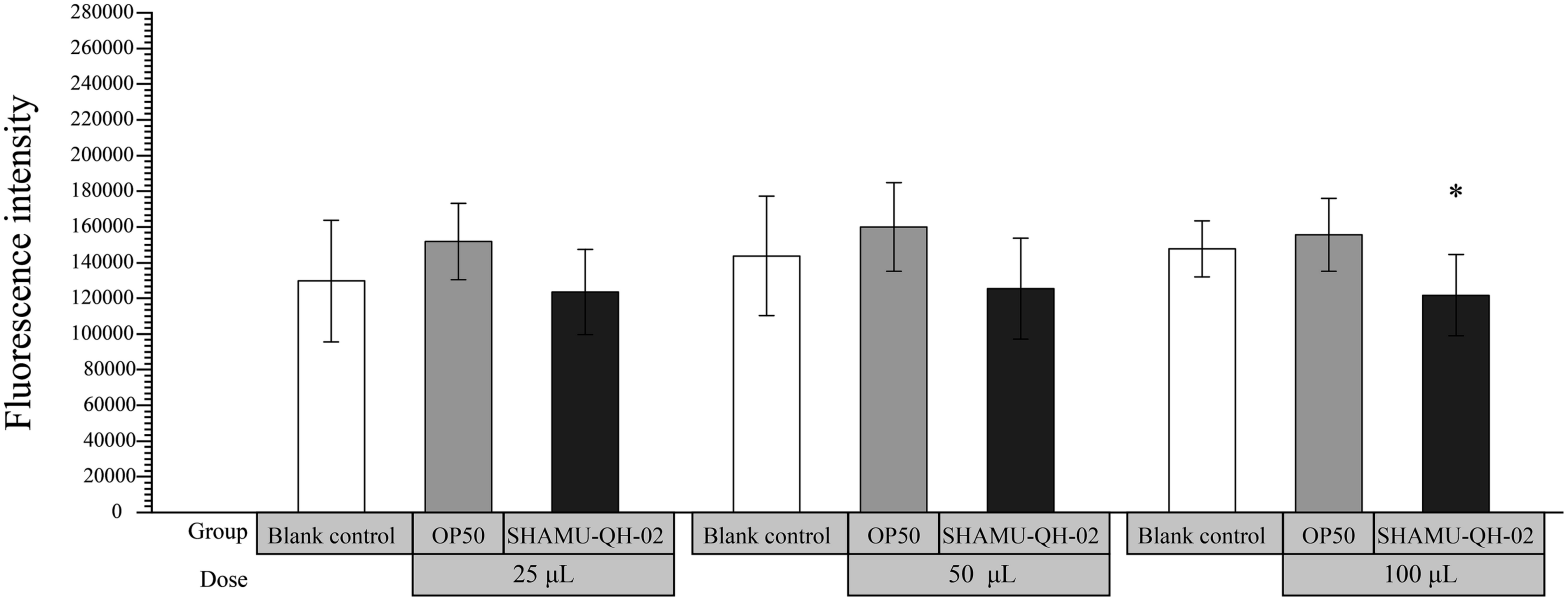
The antioxidant activity of SHAMU-QH-02 based on the scavenging ROS assay. ^*^Statistically significant versus 100 μL of *E. coli* OP50 suspension.

As shown in Figure 5, 100 μL of SHAMU-QH-02 (OD_600_ = 0.6) demonstrated significantly higher scavenging ability of ROS compared to *E. coli* OP50 (*p* = 0.039).

### 3.4 Anti-inflammatory activity

**Figure 6:**
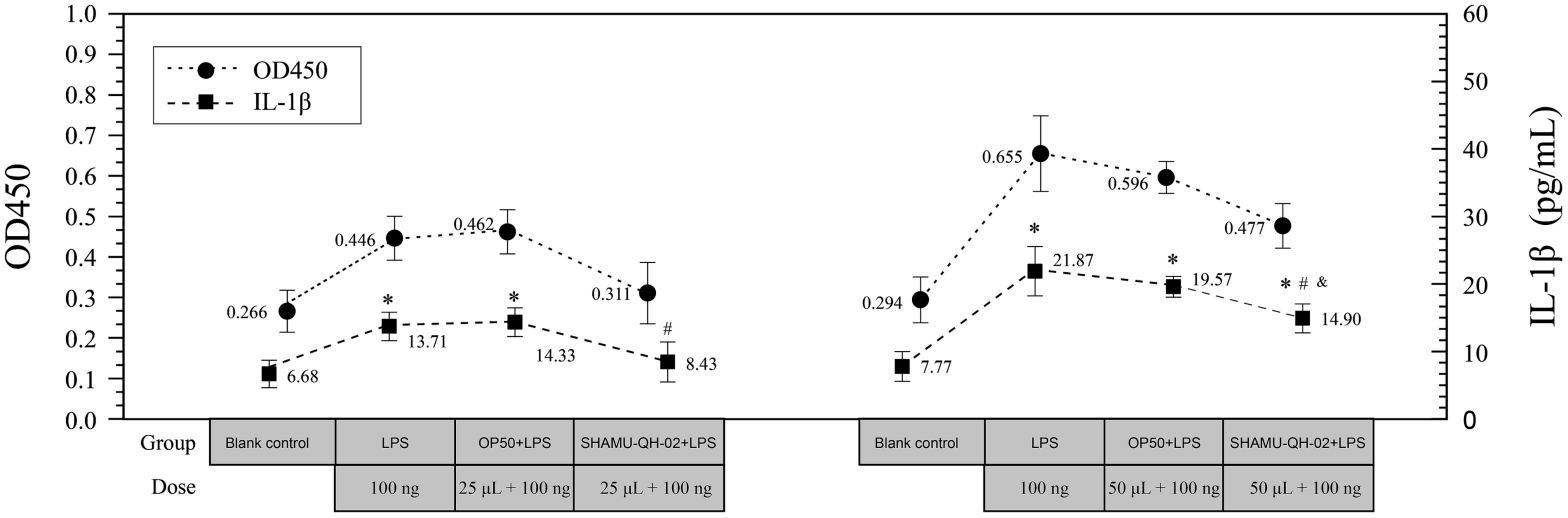
The anti-inflammatory activity of SHAMU-QH-02. ^*^Statistically significant versus blank control. ^#^Statistically significant versus *E. coli* OP50 suspension. ^&^Statistically significant versus LPS.

As shown in Figure 6, treatment with 25 μL of *E. coli* OP50 suspension did not reduce the production of IL-1β in LPS-stimulated RAW264.7 cells. In contrast, treatment with 25 μL of SHAMU-QH-02 suspension significantly decreased the production of IL-1β, reaching levels comparable to the blank baseline. In addition, treatment with 50 μL of *E. coli* OP50 suspension did not decrease the production of IL-1β. Furthermore, treatment with 50 μL of SHAMU-QH-02 suspension significantly mitigated the production of IL-1β. Taken together, these findings suggest that SHAMU-QH-02 likely secreted a bioactive substrate that could markedly decrease IL-1β production in LPS-stimulated RAW264.7 cells in a concentration-dependent manner.

### 3.5 Cytotoxicity assay

**Figure 7:**
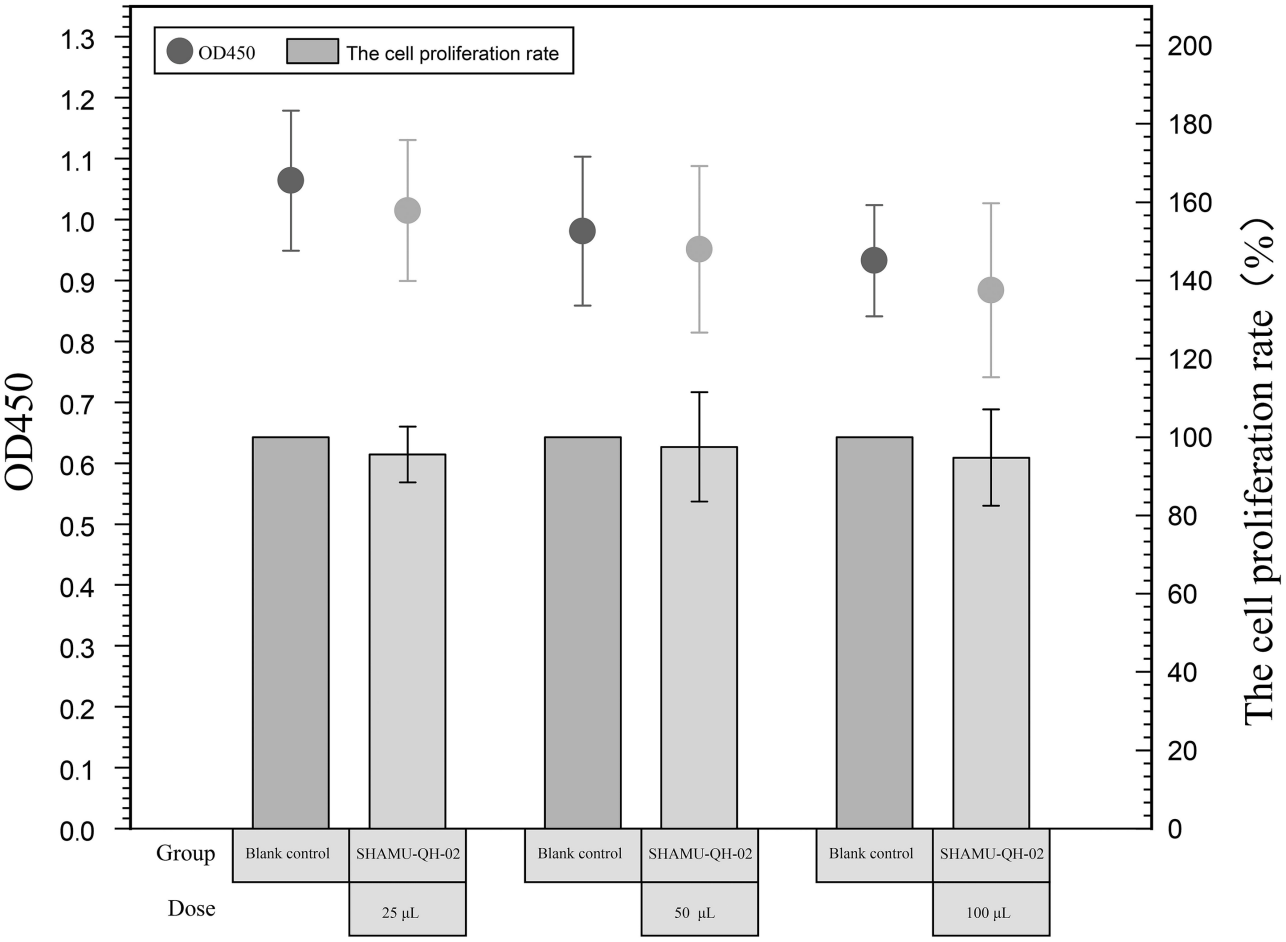
Cytotoxicity of SHAMU-QH-02.

The CCK-8 assay revealed that 25 μL, 50 μL, and 100 μL of SHAMU-QH-02 (OD_600_ = 0.6) yielded no significant effect on the proliferation of RAW264.7 macrophage cells compared with the blank control. These results indicated that SHAMU-QH-02 had no cytotoxic effects on mouse macrophage RAW264.7 cell proliferation. Refer to Figure 7 for more details.

### 3.6 Antibiotic resistance

Among the 14 antibiotics evaluated, SHAMU-QH-02 exhibited inherent resistance to VAN and QP/DP, while remaining susceptible to the remaining antibiotics tested. See Table 1 for details.

**Table 1:** Antibiotic susceptibility and resistance profile of SHAMU-QH-02.

Antimicrobial agent	Minimum inhibitory concentration (μg/mL)	Interpretation
Vancomycin	4	Resistance
Tigecycline	≤0.12	Susceptibility
Teicoplanin	≤0.5	Susceptibility
Daptomycin	0.25	Susceptibility
Penicillin	1	Susceptibility
Ampicillin	≤2	Susceptibility
Ciprofloxacin	1	Susceptibility
Levofloxacin	2	Susceptibility
Tetracycline	≤1	Susceptibility
Fosfomycin	≤64	Susceptibility
Erythromycin	0.5	Susceptibility
Linezolid	2	Susceptibility
Nitrofurantoin	≤16	Susceptibility
Quinupristin/Dalfopristin	1	Resistance

### 3.7 Gastrointestinal stability

#### 3.7.1 Acid–base and bile tolerance

**Figure 8:**
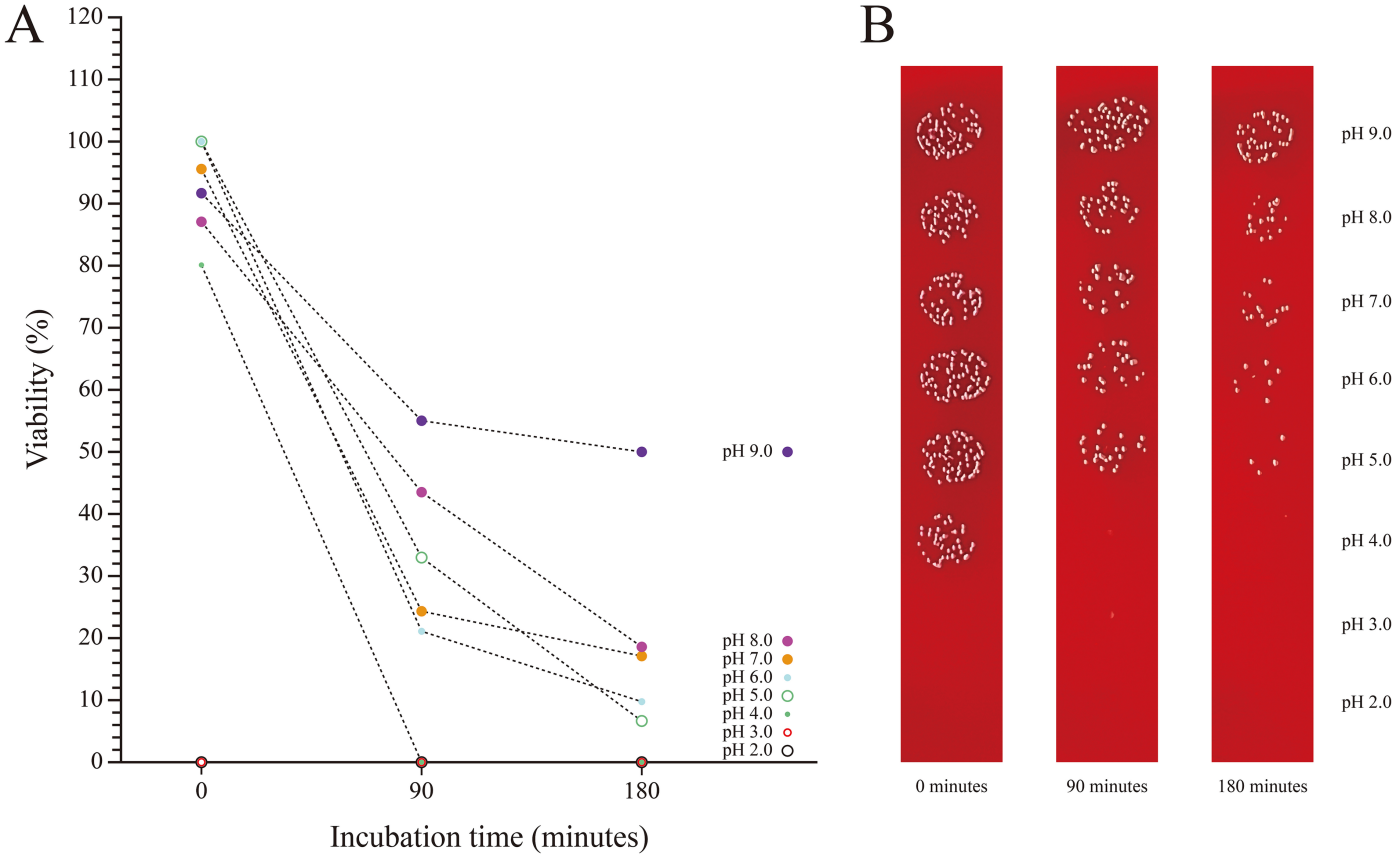
The viability of SHAMU-QH-02 under different pH conditions. **(A)** Acid-base tolerance of SHAMU-QH-02. **(B)** Viable cell counts on columbia sheep blood agar plates.

The stability of SHAMU-QH-02 under simulated gastrointestinal conditions was examined by quantifying its tolerance to acid, base and porcine bile. SHAMU-QH-02 strain demonstrated no resistance at pH 2 and pH 3. Moreover, when incubated in a pH 9 solution for 90 min, SHAMU-QH-02 strain exhibited greater survival (55.12%) compared to pH 8 (43.64%), pH 7 (24.11%), pH 6 (21.24%), pH 5 (22.39%), and pH 4 (0.00%). Similarly, after 180 min, the SHAMU-QH-02 strain still exhibited greater survival in a pH 9 solution (49.95%) compared to pH 8 (18.37%), pH 7 (17.22%), pH 6 (9.76%), pH 5 (6.32%), and pH 4 (0.00%). Refer to Figures 8A,B for more details.

**Figure 9:**
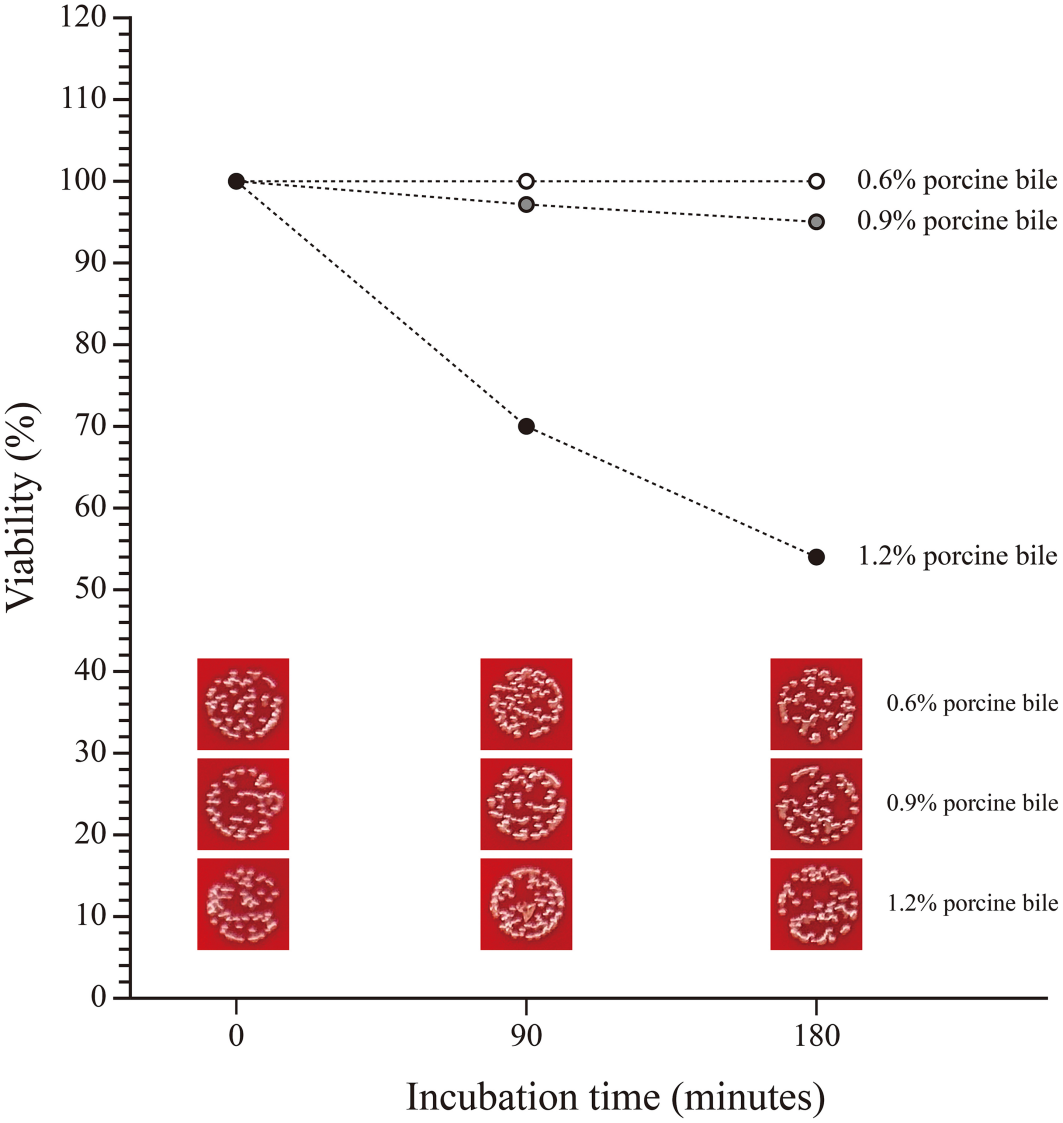
The viability of SHAMU-QH-02 under different concentrations of porcine bile.

Besides, SHAMU-QH-02 strain consistently demonstrated high tolerance to 0.6% porcine bile and 0.9% porcine bile over time, maintaining viability between 95 and 100%. In contrast, 1.2% bile negatively impacted the viability of the SHAMU-QH-02 strain to a range of 53.49–69.77%. Refer to Figure 9 for more details.

#### 3.7.2 Adhesion to intestinal epithelial cells

**Figure 10:**
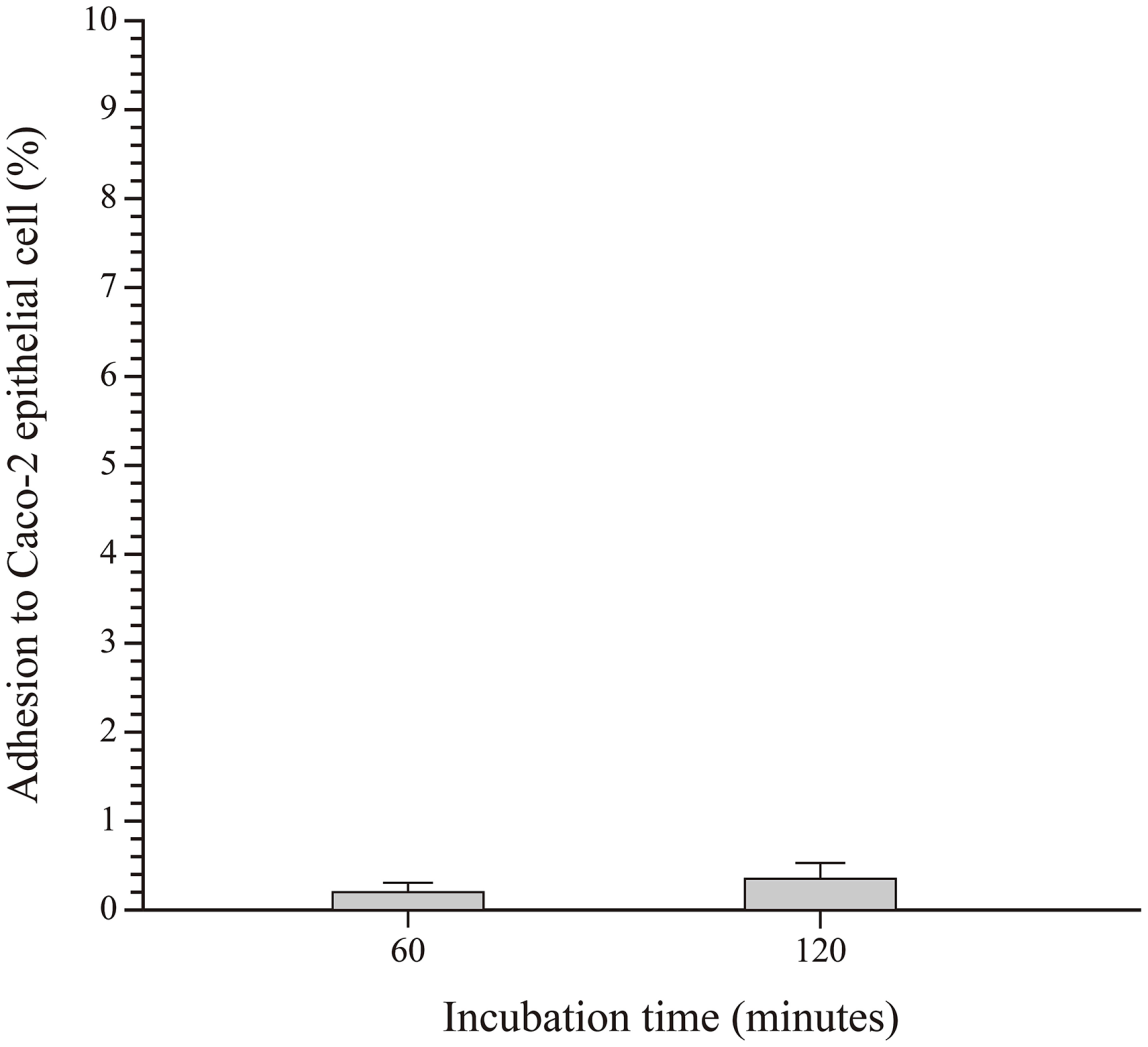
The adhesive capacity of SHAMU-QH-02 to Caco-2 cells.

The ability of SHAMU-QH-02 to adhere to intestinal epithelial cells was analyzed by incubating the strain on a Caco-2 cell monolayer (Figure 10). The results revealed comparable mean adhesion rates between incubation for 60 min (0.20 ± 0.08%) and 120 min (0.36 ± 0.12%) (*p* = 0.127).

### 3.8 Genome sequence analysis

#### 3.8.1 Bacteriocin-producing genes

**Figure 11:**
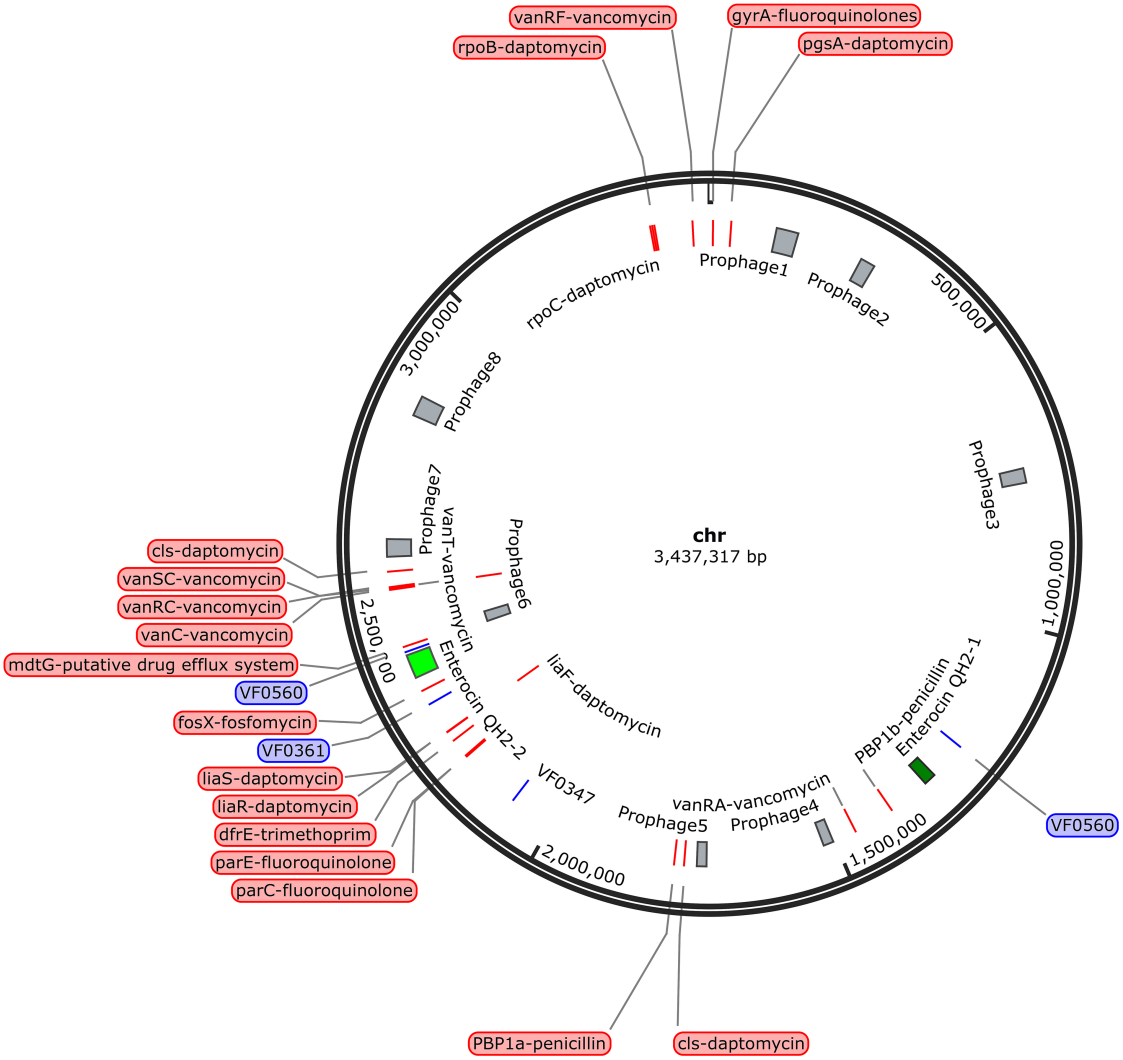
Location of the BGCs, virulence-related genes, antibiotic resistance-related genes, and phage-related genes in the SHAMU-QH-02 chromosomal genome.

By analyzing and comparing the anti-SMASH and BAGEL online databases, two putative biosynthetic gene clusters (BGCs) of enterocin QH2 were identified on the SHAMU-QH-02 chromosomal genome (Figure 11). Enterocin QH2-1 was predicted to encode terpene clusters, exhibiting only 45% homology with previously characterized carotenoid BGCs. Enterocin QH2-2 was predicted to encode type III polyketide synthases (T3PKS) clusters, exhibiting only 23% homology with viguiepinol BGCs (Table 2). These findings suggest that SHAMU-QH-02 exhibits the potential to synthesize carotenoid and viguiepinol analogs.

**Table 2:** List of two BGCs of enterocin QH2 in the SHAMU-QH-02 chromosomal genome.

Region	Type	Start	End	Most similar known cluster	Similarity
Enterocin QH2-1	Terpene	1,296,550	1,317,365	Carotenoid	45%
Enterocin QH2-2	T3PKS	2,343,619	2,384,779	Viguiepinol	23%

#### 3.8.2 Safety-related genes

To investigate the presence of virulence factors in the genome of SHAMU-QH-02, the VFDB was utilized. A total of four putative genes associated with virulence factors were identified (Figure 11). As shown in Table 3, when *Enterococcus* was specified as the genus in VFDB, genes associated with capsulation (*ugd*, *cpsB*, and *gndA*) and metabolic factor (*lplA1*) were identified. Previous research has established seven essential genes required for capsule production in *Enterococcus*: *cpsC*, *cpsD*, *cpsE*, *cpsG*, *cpsI*, *cpsJ*, and *cpsK* (Penas et al., 2013). However, in this study, the presence of *cpsB* alone did not result in capsule formation. Similarly, the production of the *Klebsiella*-related capsule involves a coordinated effort of multiple genes, with isolated *ugd* and *gndA* genes being insufficient for the biosynthesis and functional expression of the capsule. In the context of metabolic factors, the *lplA1* gene encodes a lipoate-protein ligase enzyme, instrumental in facilitating the intracellular replication of bacteria within the cytosol of host cells (Chen et al., 2017).

**Table 3:** List of virulence detected in the SHAMU-QH-02 chromosomal genome.

Identified number	Name	ORF	Start	End	VF category	Virulence factors	Related genes
*WP_004175261*	VF0560	chr_1173	1,231,632	1,232,798	Antiphagocytosis	Capsule	*ugd*
*NP_464456*	VF0347	chr_1975	2,076,064	2,077,068	Metabolic factor	LplA1	*lplA1*
*WP_002359680*	VF0361	chr_2174	2,293,039	2,293,845	Antiphagocytosis	Capsule	*cpsB*
*WP_014907233*	VF0560	chr_2272	2,390,492	2,391,913	Antiphagocytosis	Capsule	*gndA*

Regarding antibiotic resistance genes, the CARD was used. A combined total of 23 putative genes associated with antibiotic resistance were detected in the chromosome (Figure 11). Of these, there were 11 natural resistance genes related to *E. casseliflavus*, including VAN, fluoroquinolone (FQ), and trimethoprim (TMP) resistance. The remaining genes were associated with PEN resistance (*n* = 2), DAP resistance (*n* = 8), FOS resistance (*n* = 1), and the putative drug efflux system (*n* = 1) (Table 4). Notably, SHAMU-QH-02 was susceptible to PEN, DAP, and FOS in the antimicrobial susceptibility testing. Moreover, genomic analysis using PHI identified a total of eight phage regions within the chromosomal DNA (Figure 11). Importantly, the virulence-related genes and antibiotic resistance-related genes were not located within these phage sequences, suggesting a low likelihood of horizontal gene transfer to other microbes. Overall, despite the presence of genetic material in SHAMU-QH-02 that could pose potential risks, no genes were identified to negatively impact human health.

**Table 4:** List of antibiotic resistance-related genes in the SHAMU-QH-02 chromosomal genome.

Resistance genes	ORF	Start	End	Gene name
Penicillin	chr_1328	1,390,230	1,392,674	*pbp1b*
Vancomycin	chr_1385	1,461,422	1,462,099	*vanRA*
Daptomycin	chr_1,685	1,761,840	1,763,306	*cls*
Penicillin	chr_1701	1,778,844	1,781,165	*pbp1a*
Fluoroquinolone	chr_2079	2,184,256	2,186,718	*parC*
Fluoroquinolone	chr_2080	2,186,730	2,188,757	*parE*
Trimethoprim	chr_2109	2,219,201	2,220,148	*dfrE*
Daptomycin	chr_2127	2,236,952	2,237,581	*liaR*
Daptomycin	chr_2128	2,237,585	2,238,652	*liaS*
Daptomycin	chr_2129	2,238,649	2,239,380	*liaF*
Fosfomycin	chr_2205	2,319,028	2,319,438	*fosX*
Putative drug efflux system	chr_2280	2,399,772	2,400,977	*mdtG*
Vancomycin	chr_2388	2,499,372	2,500,424	*vanC*
Vancomycin	chr_2389	2,500,421	2,500,993	*vanXYC*
Vancomycin	chr_2390	2,500,990	2,503,092	*vanT*
Vancomycin	chr_2391	2,503,191	2,503,886	*vanRC*
Vancomycin	chr_2392	2,503,942	2,505,006	*vanSC*
Daptomycin	chr_2413	2,530,409	2,531,854	*cls*
Daptomycin	chr_3178	3,336,136	3,339,786	*rpoC*
Daptomycin	chr_3179	3,339,874	3,343,491	*rpoB*
Vancomycin	chr_3240	3,408,518	3,409,207	*vanRF*
Daptomycin	chr_38	37,244	37,825	*pgsA*
Fluoroquinolone	chr_6	6,168	8,693	*gyrA*

### 3.9 Stability of crude extract to pH, temperature, and proteases

**Figure 12:**
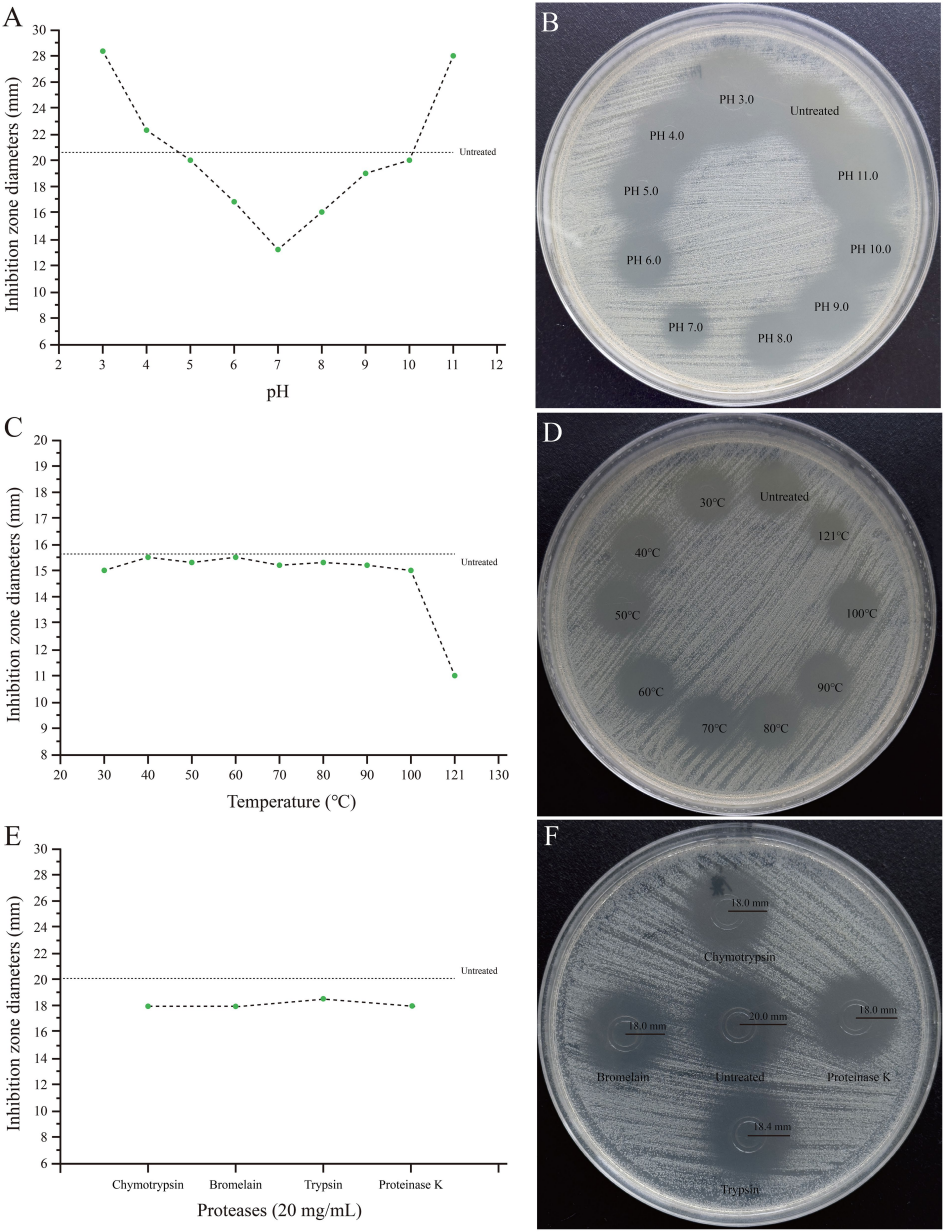
Stability of the crude extract of SHAMU-QH-02 to pH, temperature, and proteases. **(A,B)** Inhibition zones across different pH levels. **(C,D)** Inhibition zones across different temperatures. **(E,F)** Inhibition zones across different proteases.

The results confirmed that the active ingredients were predominantly present in 80 and 90% ethanol eluates. The crude extract of SHAMU-QH-02 maintained its antimicrobial activity over a relatively wide pH range (3.0 to 11.0). Interestingly, the antibacterial activity of the crude extract was attenuated at near-neutral pH levels (6.0 to 8.0), similar to the untreated group at pH 4.0, pH 5.0, pH 9.0, and pH 10.0, but enhanced at extreme pH levels (Figures 12A,B). The crude extract demonstrated resistance to heat treatment at 121°C, retaining most of the antibacterial activity (Figures 12C,D). The crude extract maintained antimicrobial activity after being treated with chymotrypsin, bromelain, trypsin, or proteinase K (Figures 12E,F).

## 4 Discussion

Currently, there remains a need to discover novel, high-quality probiotics and related products to meet complex application requirements across various fields. Although previous studies have revealed several potential probiotic attributes of *E. casseliflavus* strains isolated from chicken, deer, snail, and common carp (Akbari et al., 2021; Carvajal et al., 2023; Rabaoui et al., 2023; Hameed and Nazir, 2024), little is known about the probiotic properties and beneficial effects of *E. casseliflavus*, especially derived from human sources. To our knowledge, SHAMU-QH-02 represents the first probiotic *E. casseliflavus* strain isolated from a human source, suggesting its potential as a probiotic supplement for human use.

Previous studies have demonstrated that *E. casseliflavus* JBL1056 and IM 416K1 exhibited robust bactericidal activity against *L. monocytogenes*, as well as partial activity against *E. faecalis* and *E. faecium* (Arihara et al., 1993; Sabia et al., 2002, 2004). Subsequently, *E. casseliflavus* strains from rangeland cattle and American bison displayed antagonistic activity against *Streptococcus pyogenes*, *S. aureus*, and *E. faecalis* (Anderson et al., 2008). Moreover, an *E. casseliflavus* strain isolated from surface water exhibited partial antagonism to *Acinetobacter* spp., *E. faecalis*, and *E. faecium* (De Niederhäusern et al., 2013). However, the SHAMU-QH-02 strain demonstrated a broader antimicrobial spectrum (encompassing most gram-positive cocci, gram-positive bacilli, and gram-negative bacilli), surpassing that of strains reported previously. Notably, the SHAMU-QH-02 strain exhibited antagonistic activity against notorious foodborne pathogens, such as *B. cereus*, *L. monocytogenes*, *S. enteritidis*, *V. fluvialis*, *E. coli*, *P. aeruginosa*, and *S. aureus*. These pathogens are known to cause food poisoning and fatal invasive infections in susceptible animals and humans, thereby posing a serious threat to public health (Ayyash et al., 2020). Notably, the crude extract of the SHAMU-QH-02 strain demonstrated sustained antimicrobial activity across a broad pH range, exhibiting resistance to temperatures of 121°C and proteolytic degradation. The stability of hypothetical enterocin QH2 surpassed that of enterocin 416K1, an antilisterial bacteriocin produced by *E. casseliflavus* IM 416K1 isolated from Italian sausages (Sabia et al., 2002).

Oxidative stress and inflammation produce a wide variety of bioactive chemicals, including pro-inflammatory cytokines, ROS, and nitrogenous products (Firdous et al., 2015). They play a significant role in the progression of several diseases, such as cancer, atherosclerosis, diabetes, obesity, and Alzheimer’s disease. Notably, probiotics exhibit antioxidant and anti-inflammatory effects by releasing specific antimicrobial substances, regulating the immune system, enhancing the antioxidant response, and reducing free radical production (Wang et al., 2017; Roy and Dhaneshwar, 2023). To our knowledge, no study has hitherto reported on the antioxidant and anti-inflammatory properties of *E. casseliflavus*. In the present study, we provided compelling evidence that the SHAMU-QH-02 strain harbored strong ABTS^+^ and ROS scavenging ability. Besides, the SHAMU-QH-02 strain could markedly decrease the production of IL-1β in LPS-stimulated RAW264.7 cells in a concentration-dependent manner. Furthermore, suspected terpene and T3PKS BGCs of enterocin QH2 were identified by gene analysis, indicating the SHAMU-QH-02 strain might have the potential to synthesize carotenoid and viguiepinol analogs. Terpenoids and related products catalyzed by T3PKS have attracted extensive attention for their antimicrobial, antioxidant, anti-inflammatory, antiviral, or anticancer activities. According to their chemical skeletons, terpenoids can be divided into monoterpenoids, sesquiterpenoids, diterpenoids, sesterterpenes, triterpenoids and polyterpenoids (Ge et al., 2022). Notably, carotenoids are tetraterpenoids, while viguiepinol belongs to the diterpenoids. This compositional diversity may contribute to the observed antimicrobial, antioxidant, and anti-inflammatory probiotic properties exhibited by the SHAMU-QH-02 strain.

The potential probiotic application of the SHAMU-QH-02 strain necessitates a comprehensive safety evaluation. In the present study, the SHAMU-QH-02 strain did not demonstrate overt cytotoxicity to RAW264.7 macrophage cells and was susceptible to 12 common antibiotics. The virulence factors of *Enterococcus* encompassed cytolysin (*cylA*, *cylB*, *cylI*, *cylL-l*, *cylL-s*, *cylM*, *cylR1*, and *cylR2*), enterococcal surface proteins (*esp*), aggregation substances (*asa1*, *asp1*, *agg*), hyaluronidase (*hyl*), and gelatinase (*gelE*) (Kim et al., 2022). In our study, genomic analysis identified four virulence-related genes, 23 antibiotic resistance-related genes, and eight phage regions in the SHAMU-QH-02 genomes. Among these, *cpsB* was involved in the biosynthesis of *enterococcal* capsular polysaccharides. However, the presence of *cpsB* alone was insufficient for the biosynthesis and functional expression of the capsule. Regarding antibiotic resistance genes, the SHAMU-QH-02 strain harbored 11 natural resistance genes (seven for VAN, three for FQ, one for TMP), two PEN resistance genes, eight DAP resistance genes, one FOS resistance gene, and one putative drug efflux system gene. Notably, the SHAMU-QH-02 strain was susceptible to PEN, DAP, and FOS in the antimicrobial susceptibility testing. Moreover, the virulence-related genes and antibiotic resistance-related genes were not located within the eight phage-related genes, suggesting a low probability of horizontal gene transfer. Taken together, these findings indicate that the SHAMU-QH-02 strain presents an acceptably low safety risk profile for potential applications.

Indeed, probiotics are expected to possess the ability to withstand bile and adhere to intestinal epithelial cells. In this study, the SHAMU-QH-02 strain demonstrated tolerance to pH 7 and pH 8 solutions, and 1.2% porcine bile *in vitro*. These results confirmed that the SHAMU-QH-02 strain could survive well in the intestine. However, the SHAMU-QH-02 strain demonstrated a lack of resistance to gastric juice (pH 2 to 3) and a limited ability to adhere to Caco-2 intestinal epithelial cells. Despite these limitations, the SHAMU-QH-02 strain could be protected from the extreme pH of gastric juice and exert its probiotic activity in the intestine by formulating it into enteric-coated or controlled-release tablets/capsules, or by adjusting the dosage and frequency of administration.

Nonetheless, it is undeniable that the present study has some noteworthy limitations. Firstly, the presence of virulence and antibiotic resistance genes may raise safety concerns, even in the absence of phenotypic expression *in vitro*. Secondly, our *in vitro* findings require validation through *in vivo* studies for a more comprehensive safety and efficacy assessment. Thirdly, given the small number and clinical origin of the strains tested, additional studies using larger number of reference strains and clinical strains will be needed to evaluate the antimicrobial activity. Fourthly, because *E. casseliflavus* is a common gut commensal, strains with superior probiotic value to the SHAMU-QH-02 are theoretically discoverable.

## 5 Conclusion

The SHAMU-QH-02 strain demonstrates probiotic characteristics, including antagonistic activity, antioxidant activity, anti-inflammatory activity, safety, and intestinal stability. The strain likely secretes two novel bacteriocins with a broad antimicrobial spectrum and stable activity, presenting as a notably promising candidate for the food industry, health promotion and disease prevention. However, further investigations are warranted to assess its persistence in the animal or human gastrointestinal tract, as well as the mechanisms underlying antimicrobial activities.

## Data Availability

The raw data supporting the conclusions of this article will be made available by the authors, without undue reservation.

## Footnotes

^1^ https://blast.ncbi.nlm.nih.gov/Blast.cgi

^2^ https://www.megasoftware.net/

^3^ https://itol.embl.de/
